# Electroconvulsive therapy increases temporarily plasma vascular endothelial growth factor in patients with major depressive disorder

**DOI:** 10.1002/brb3.2001

**Published:** 2021-08-02

**Authors:** Annamari Sorri, Kaija Järventausta, Olli Kampman, Kai Lehtimäki, Minna Björkqvist, Kati Tuohimaa, Mari Hämäläinen, Eeva Moilanen, Esa Leinonen

**Affiliations:** ^1^ Department of Psychiatry Tampere University Hospital Tampere Finland; ^2^ Faculty of Medicine and Health Technology Tampere University Tampere Finland; ^3^ Department of Neurosurgery, Neurology and Rehabilitation Tampere University Hospital Tampere Finland; ^4^ The Immunopharmacology Research Group Faculty of Medicine and Health Technology Tampere University and Tampere University Hospital Tampere Finland

**Keywords:** electroconvulsive therapy, major depressive disorder, **v**ascular endothelial growth factor

## Abstract

**Objectives:**

Vascular endothelial growth factor (VEGF) has been related to the etiology of major depressive disorder (MDD). The findings involving the effects of electroconvulsive therapy (ECT) on the VEGF levels have been conflicting. The aim was to examine the possible changes in the VEGF levels and their associations with clinical outcome in patients with MDD during ECT.

**Methods:**

The study comprised 30 patients suffering from MDD. Their plasma VEGF levels were measured at baseline and 2 and 4 hr after the first, fifth, and last ECT session. The severity of depression was quantified by the Montgomery‐Asberg Depression Rating Scale (MADRS).

**Results:**

The VEGF levels increased between the 2‐hr and 4‐hr measurements during the first (*p* = .003) and the fifth (*p* = .017) sessions. The baseline VEGF levels between individual ECT sessions remained unchanged during the ECT series. No correlations were found between the increased VEGF levels and the clinical outcome.

**Conclusions:**

Electroconvulsive therapy increased the VEGF levels repeatedly at the same time point in two different ECT sessions. These increases had no association with the response to ECT. Consequently, VEGF may act as a mediator in the mechanism of action of ECT.

## Graphical abstract

Plasma VEGF levels of patients suffering from MDD were measured in different time points during ECT series. ECT increased the VEGF levels repeatedly at the same time point in two different ECT sessions but these increases had no association with the response to ECT. Consequently, VEGF may act as a mediator in the mechanism of action of ECT.

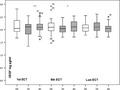

## 1 INTRODUCTION

Electroconvulsive treatment (ECT) is considered to be among the most effective treatments for severe depression (UK ECT Review Group, 2003), yet the mechanisms of its action are not fully understood. ECT is used for major depressive disorders (MDD) when a rapid improvement is needed because of severe psychotic or suicidal symptoms or for treatment‐resistant depression (American Psychiatric Association, 2009; National Institute for Health and Clinical Excellence (NICE), 2009).

Vascular endothelial growth factor (VEGF) was first presented as an inducer of vascular permeability (Dvorak, 2006; Leung et al., 1989), and subsequently, the understanding of its actions has broadened to include angiogenesis and vasculogenesis stimulation (Millauer et al., 1993) as well as its role as a promoter of neurogenesis and neuroprotective mechanisms (Jin et al., 2002; Storkebaum et al., 2004). Moreover, it has been suggested that VEGF has an effect on alterations in cognitive function and behavior due to its direct neurogenic actions (Cao et al., 2004). The neurotrophic hypothesis of MDD postulates that stress induces alterations in neuroplastic pathways in brain areas accounting for emotional and cognitive processing, leading to reduced neurogenesis and hippocampal volume, predisposing to depression. Although the underlying pathophysiological mechanisms of MDD as a whole are currently only partly understood, growing evidence has demonstrated that the etiology and treatment of MDD might be related to the molecular and volume changes in the hippocampus (Fournier & Duman, 2012). These alterations appear to be mediated by decreased expression of neurotrophins, such as brain‐derived neurotrophic factor (BDNF) and subsequently also VEGF (Duman, R. S. & Monteggia, 2006; Duman et al., 1997).

In rodent models, electroconvulsive seizures (ECS) have been reported to stimulate hippocampal neurogenesis (Madsen et al., 2000; Malberg et al., 2000). Furthermore, ECS induces VEGF expression in the hippocampus (Altar et al., 2004; Elfving & Wegener, 2012; Newton et al., 2003).

In spite of certain inconsistencies concerning the materials as well as the methodologies in VEGF studies, several meta‐analyses have reported higher blood VEGF levels in patients with MDD compared with healthy controls. However, opposite findings have also been reported, indicating no significant differences between patients with depression and nondepressed controls (Carvalho et al., 2015; Clark‐Raymond et al., 2014; Sharma et al., 2016; Tseng et al., 2015). It has been hypothesized that the inconsistencies between these results originate from the characteristics of MDD, which is now considered to be a heterogeneous cluster of subgroups including various individual traits of symptoms rather than a specific consistent syndrome (Fried & Nesse, 2015). Different subtypes of MDD have been described based on symptom presentations, treatment responsiveness, or different underlying neurobiological mechanisms to explain the heterogeneity between the studies and to determine specific treatments. However, an understanding of the MDD subtypes based on the findings of the studies is still insufficient (Kunugi et al., 2015; Strawbridge et al., 2017). In studies on treatment‐resistant depression (TRD), the VEGF results have also been contradictory (Carvalho, L. A. et al., 2013; Pisoni et al., 2018; Viikki et al., 2010). Decreased plasma and cerebrospinal fluid VEGF levels have been associated with suicide risk in suicide attempters (Isung, Mobarrez et al., 2012; Isung, Aeinehband et al., 2012).

The possible influence of antidepressants on blood VEGF levels and their association with treatment response has also been studied with the aim of finding a potential predictor for treatment response. Also, these reports have been contradictory regarding both the blood VEGF levels and the relation between VEGF levels and treatment outcomes (Buttenschøn et al., 2015; Clark‐Raymond et al., 2017; Fornaro et al., 2013; Halmai et al., 2013; Pisoni et al., 2018; Ventriglia et al., 2009).

In patients with depression, the effects of ECT on blood VEGF levels have appeared to be somewhat variable. Minelli et al. (Minelli et al., 2011, 2014) have reported an increase in VEGF levels after the ECT series, whereas in another study, the blood VEGFA mRNA levels decreased after the treatment series in patients with psychotic depression (Kolshus et al., 2017). In a recent study, no overall changes were found (Ryan & McLoughlin, 2018). Moreover, no changes in cerebrospinal fluid (CSF) VEGF levels were found after the ECT series (Kranaster et al., 2019).

Because the current data concerning the possible influence of ECT on VEGF levels are scarce and somewhat contradictory, the aim of the present study was to examine the possible effects of ECT on VEGF plasma levels and their associations with clinical outcomes in patients with MDD. Three time points (the first, the fifth, and the last ECT) were targeted to study the acute and the long‐term effects.

## 2 MATERIALS AND METHODS

Forty‐nine consecutive patients with severe or psychotic MDD were voluntarily asked to participate in the present study while admitted for ECT in the Department of Psychiatry, Tampere University Hospital. Thirteen patients declined, and six patients discontinued the study. Consequently, 30 patients (12 female and 18 male) were included in the study. The diagnosis of major depressive disorder (MDD) was confirmed with each patient by using the Structured Clinical Interview for DSM‐IV Disorders (SCID) (First et al., 1996), and 14 patients presented psychotic symptoms. Nine patients suffered their first episode of MDD, and 21 patients suffered from recurrent MDD.

The exclusion criteria were progressive organic brain disorder, major psychiatric disorders other than MDD, inflammatory or autoimmune disease, epilepsy, alcohol or other substance use, and previous ECT within three months of the start of the study.

The mean age of the patients was 57.1 years (*SD* 17.7, range 25–85 years). Patients were maintained on their psychotropic medication during the ECT series (Table 1). Twenty‐eight patients were treated with a combination of at least two psychotropic medications and two were on monotherapy. To avoid effects on the seizure threshold, the benzodiazepines were intermitted ten hours before each ECT.

**Table 1:** Clinical characteristics and psychotropic medications of patients with MDD during the ECT series

	All patients (*n* = 30)	Female patients (*n* = 12)	Male patients (*n* = 18)
Age, mean, ±*SD*	57.1, ± 17.7	71.1 ± 12.2	45.2 ± 17.0
range	25–85	45–85	25–79
Total number of ECTs, mean, ±*SD*	10.4, ± 3.6	10.8 ± 4.3	8.2 ± 3,6
range	5–17	5–17	5–13
Psychotic symptoms	14	7	7
First episode of MDD	9	4	5
Recurrent MDD	21	8	13
Antidepressants			
SSRI	9	3	6
SNRI	12	5	7
Mirtazapine	12	6	6
Bupropion	4	1	3
Antipsychotics			
First‐generation antipsychotics	1	0	1
Second‐generation antipsychotics	28	11	17
Anxiolytics			
Benzodiazepines	21	8	13
Pregabalin	2	2	0
Buspirone	1	0	1

Note

Abbreviations: SNRI, Serotonin and norepinephrine reuptake inhibitor; SSRI, Selective serotonin reuptake inhibitor.

Depression severity was assessed using the Montgomery‐Asberg Depression Rating Scale (MADRS) (Montgomery & Åsberg, 1979) before the ECT series and after the fifth and last ECT.

A medical history and a physical examination with routine blood examinations and end electrocardiogram (ECG) were requested before the ECT series. ECT was performed with a brief pulse constant current device, the MECTA SPECTRUM 5000Q (MECTA Corp., Lake Oswego, OR, USA). Anesthesia for ECT was induced by intravenous (i.v.) methohexital (initial dose 1 mg/kg), and muscle relaxation was achieved with i.v. succinylcholine (initial dose 0.5 mg/kg). The patients were ventilated with 100% oxygen until restart of spontaneous respiration. During the ECT treatment pulse oximetry, blood pressure, ECG, one channel electroencephalogram (EEG), and electromyography (EMG) were monitored.

Patients were treated three times a week with standard bilateral ECT. Seizure threshold was titrated during the first ECT session and subsequent treatments were administered at 1.5 times the seizure threshold. Seizures over 25 s in duration in the electroencephalogram (EEG) were defined adequate. If the seizure was insufficient, the stimulation dose was increased subsequently. The number of treatments ranged between 5 and 17, 10.4 ± 3.6 (mean ± *SD*). ECT treatment was discontinued on the basis of clinical judgment if the patient was either in remission (MADRS ≤ 10) or no further improvement was recorded during the last two ECT sessions. In five patients, the fifth ECT was the last (not included in the last ECT session data).

Blood was drawn before ECT (baseline) and two hours and four hours after ECT at the first, fifth, and last session. EDTA‐treated plasma was separated and stored at −80°C until analyzed. The concentration of VEGF was determined by enzyme‐linked immunosorbent assay with commercial reagents (Quantikine; R&D Systems Europe, Ltd., Abingdon, UK). The detection limit and interassay coefficient of variation were 7.8 pg/ml and 7.3%, respectively.

This study design was reviewed and approved by the Tampere University Hospital Ethics Committee. Written informed consent was obtained from each patient.

## 3 STATISTICAL METHODS

The data were tested for normality using Q–Q plots. Because the distributions were non‐normal, logarithmic transformations were used for plasma VEGF values in all analyses. A paired sample *t* test was used to analyze changes between the VEGF level at different measurement points: baseline versus. 2‐hr, baseline versus. 4‐hr, and 2‐hr versus. 4‐hr. Correlational analyses of the relationships between the VEGF levels and symptom reduction according to the MADRS were done using Pearson correlation coefficients (Pearson's r) and the paired sample *t* test. The level of statistical significance was set at *p* < .05, except for changes in the VEGF level at different measurement points a Bonferroni correction was used due to multiple testing (a total of 9 tests, Bonferroni‐corrected significance *p* < .0056).

## 4 RESULTS

The mean MADRS score at baseline was 31.6 ± 7.2 (mean ± *SD*). After the ECT series, the mean score was 11.3 ± 7.5. At the end of the study, 22 patients out of 30 (73.3%) showed at least a 50% decrease in their MADRS score (response). Twenty patients out of 30 (66.7%) were in remission (MADRS ≤ 10).

**Figure 1:**
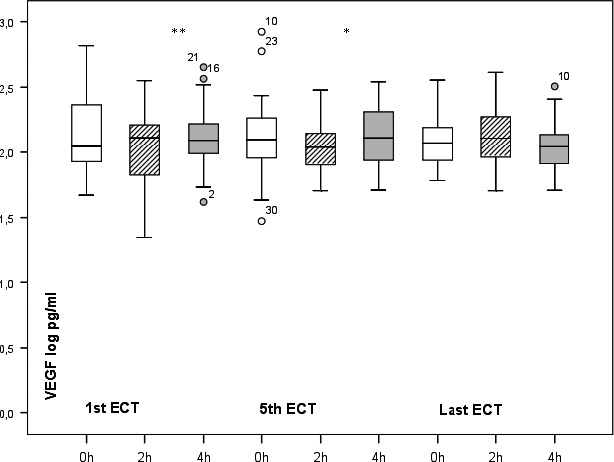
Plasma VEGF log levels of the first, the fifth and the last ECT sessions

The medians, quartiles, and interquartile ranges of plasma VEGF during the first, fifth, and last ECT sessions are presented in Table 2. There was an increase in the plasma VEGF levels between the 2‐hr and 4‐hr measurement points during the first (*p* = .003) and fifth (*p* = .017) ECT sessions (Figure 1). During the last ECT session, this change in VEGF levels between 2‐hr and 4‐hr was not significant. There were no significant changes in the levels of VEGF between the baseline and 2‐hr or the baseline and the 4‐hr measurements at any studied ECT sessions. When comparing the baseline plasma VEGF levels in the first, fifth, and last ECT sessions, no significant changes were found in the whole study group (Figure 1). The increases found in plasma VEGF levels between 2‐hr and 4‐hr measurements during the first and fifth ECT sessions did not correlate with the MADRS relative response (r = –0.182, *p* = .335 and r = –0.163, *p* = .390, respectively).

**Table 2:** Medians, quartiles, and interquartile ranges of plasma VEGF during the first, fifth, and last ECT sessions and statistical values of the paired sample *t* tests

ECT	*n*	Median (pg/ml)	First quartile (pg/ml)	Third quartile (pg/ml)	IQR (pg/ml)	Paired comparisons (0–2 and 2–4 hr)	
						*t*	*P*
1st ECT baseline	30	107.05	78.88	204.05	125.18		
2‐hr	30	100.70	65.83	157.98	92.15	1.86	0.073
4‐hr	30	123.45	92.43	175.20	82.78	−3.27	0.003
5th ECT baseline	30	128.80	89.48	187.88	98.40		
2‐hr	30	105.95	83.10	148.20	65.10	1.53	0.14
4‐hr	30	126.80	88.50	206.23	117.73	−2.53	0.017
last ECT baseline	25	118.30	82.25	162.15	79.90		
2‐hr	25	128.80	88.55	190.80	102.25	−0.92	0.37
4‐hr	25	110.20	80.30	150.50	70.20	1.25	0.22

Abbreviation: IQR, interquartile range.

* In the comparisons between 0–4 hr, *p* = .48, *p* = .70, and *p* = .74 at first, 5th and last ECT, respectively

## 5 DISCUSSION

In the present study, an increase in plasma VEGF levels between the 2‐hr and 4‐hr measurement points was found during the first and the fifth sessions. However, given the total number of analyses made, the latter difference was only nominally significant. The baseline levels of plasma VEGF levels remained unaltered in different ECT sessions. No correlations were found between the observed increases in plasma VEGF levels and the clinical outcomes. To our knowledge, the present study is the first to examine the changes in plasma VEGF levels during an ECT series. In rodents, the VEGF levels were increased up to 3h after ECS and began to decrease after 6 hr (Elfving & Wegener, 2012). The chosen timings (sampling at 2h and 4h) would be in line with these findings. The increases found in plasma VEGF levels might be a repetitive phenomenon, since the changes seem to have appeared in two different ECT sessions at the same time point.

Recent studies have reported increased volume and morphological changes in the hippocampus and the amygdala induced by the ECT (Joshi et al., 2016; Nordanskog et al., 2010; Tendolkar et al., 2013). VEGF appears to promote hippocampal neurogenic effects, both indirectly by stimulating endothelial cell production and the release of key neurotrophic factors, and directly by enhancing mitogenic effects on neuronal precursors (Fournier & Duman, 2012). The increase in plasma VEGF during the ECT series could suggest that VEGF is acting as some kind of mediator in the process stimulated by ECT.

In previous studies, the blood VEGF levels were measured after the ECT series. Minelli et al. (2011) assessed the serum VEGF levels the day after and one month after the end of the ECT series and reported a significant increase in the serum VEGF levels between the baseline and one month after the ECT series. Additionally, there was a correlation between the increased VEGF levels and a reduction in the depressive symptoms (Minelli et al., 2011). Kolshus et al. (2017) compared both whole blood VEGF and VEGF mRNA levels at baseline and 1–3 days after the ECT series and observed a decrease in their levels in patients with psychotic depression (Kolshus et al., 2017). A recent study explored the plasma VEGF levels before the first ECT and 1–3 days following the ECT series and no changes were detected (Ryan & McLoughlin, 2018). The timing of blood samplings and other methodological differences between these studies and the present one hinders the comparison between them (Kolshus et al., 2017; Minelli et al., 2014; Ryan & McLoughlin, 2018). In the CSF, the baseline VEGF levels were reported to be lower in patients with MDD compared with healthy controls, whereas no changes were found in the CSF VEGF levels measured one and seven days post‐ECT when compared to the baseline (Kranaster et al., 2019).

Reports on the associations between the blood VEGF levels and MDD have been contradictory, although most studies reported high blood VEGF levels in patients with MDD (Sharma et al., 2016). Furthermore, the range of plasma VEGF levels has varied extensively between the different studies, making a comparison impossible. In the present study, the range of levels of VEGF was of the same magnitude as the levels reported by Takebayashi et al. (2010) and Dome et al. (2012) (Dome et al., 2009; Dome et al., 2012; Takebayashi et al., 2010). Some diurnal variation in VEGF levels have been reported (Hanefeld et al., 2017; Hetland et al., 2008). However, all the present baseline samples were taken between at 8.30 and 10.30 a.m. at the same time as the ECT was administered. Peripheral VEGF levels are highly sensitive to external/extrinsic factors involved in the sampling process; for example, the type of anticoagulant used to collect blood, blood collection tube type (glass or plastic), precentrifugation time delay, and time delay for storage (Ryan & McLoughlin, 2018).

The limitations of the present study include the proportionately small sample size. The number of patients was somewhat smaller (*n* = 25) at the last sampling, which may contribute to the finding of this ECT session. Furthermore, the interpretation of the results would have benefitted from the VEGF levels of healthy controls and the measurement of VEGF levels after a period of time subsequent to the ECT series. The differences between the methodologies in the previous studies concerning the association of ECT and blood VEGF levels hamper the comparison with the present results. The present population include both sexes and a wide age range. However, only minimal differences in VEGF levels between the sexes have been reported (Meo et al., 2005; Walz et al., 2016). The patients ages would not have affected the present results because the range of ages remained the same throughout the study. Another limitation is the uncertainty over how accurately plasma VEGF levels reflect VEGF levels in the brain. Elfving et al. (2010) implied that peripheral VEGF levels might not be associated with the VEGF levels in the brain (Elfving et al., 2010). Also, whether this increase in the VEGF levels found in the present study is related to the anesthetics used or to the mechanisms of ECT is unclear.

The strengths of the present study include the careful evaluation of the patients, the standardized and systematic timing of blood sampling after the separate ECT sessions and throughout the ECT series, which enhances the reliability of the result. The samples were assayed concurrently; hence, they are comparable.

In conclusion, the VEGF levels increased between the 2‐hr and 4‐hr measurements after a single ECT. This increase had no correlation with the response to treatment. Thus, VEGF may act as a mediator in the mechanism of ECT.

## CONFLICTS OF INTEREST

None declared.

## AUTHOR CONTRIBUTION

E.L., K.L., K.J., M.H., E.M., and A.S. conceived and designed the study, K.J., K.T., M.B., and A.S. enrolled the patients and K.T. and M.B. did the blood sampling. M.H. and E.M. were in charge of the sample analysis. O.K. analyzed the data. A.S. and E.L wrote the manuscript. All authors read and approved the final manuscript.

### PEER REVIEW

The peer review history for this article is available at https://publons.com/publon/10.1002/brb3.2001.

## Data Availability

The data that support the findings of this study are available from the corresponding author upon reasonable request.
